# Synthesis, characterization, and growth simulations of Cu–Pt bimetallic nanoclusters

**DOI:** 10.3762/bjnano.5.150

**Published:** 2014-08-27

**Authors:** Subarna Khanal, Ana Spitale, Nabraj Bhattarai, Daniel Bahena, J Jesus Velazquez-Salazar, Sergio Mejía-Rosales, Marcelo M. Mariscal, Miguel José-Yacaman

**Affiliations:** 1 Department of Physics and Astronomy, University of Texas at San Antonio, One UTSA Circle, 78249, San Antonio, Texas, USA; 2 INFIQC, CONICET, Departamento de Matemática y Física, Facultad de Ciencias Químicas, Universidad Nacional de Córdoba, (XUA5000) Córdoba, Argentina; 3 Center for Innovation and Research in Engineering and Technology, and CICFIM-Facultad de Ciencias Físico-Matemáticas, Universidad Autónoma de Nuevo León, San Nicolás de los Garza, NL 66450, México

**Keywords:** Cu–Pt clusters, energy dispersive X-ray spectroscopy (EDX), grand-canonical Langevin dynamics, nanoalloys, scanning transmission electron microscopy (STEM)

## Abstract

Highly monodispersed Cu–Pt bimetallic nanoclusters were synthesized by a facile synthesis approach. Analysis of transmission electron microscopy (TEM) and spherical aberration (*C*_s_)-corrected scanning transmission electron microscopy (STEM) images shows that the average diameter of the Cu–Pt nanoclusters is 3.0 ± 1.0 nm. The high angle annular dark field (HAADF-STEM) images, intensity profiles, and energy dispersive X-ray spectroscopy (EDX) line scans, allowed us to study the distribution of Cu and Pt with atomistic resolution, finding that Pt is embedded randomly in the Cu lattice. A novel simulation method is applied to study the growth mechanism, which shows the formation of alloy structures in good agreement with the experimental evidence. The findings give insight into the formation mechanism of the nanosized Cu–Pt bimetallic catalysts.

## Introduction

The study of bimetallic (BM) nanoclusters has received particular interest because of their myriad properties and applications in optics, magnetism, catalysis, and others, mainly because their high tunability and superior features compared with those of their monometallic counterparts [1–6]. Depending on the elements, relative concentrations, and details of the synthesis method, the BM may form core–shell structures, heterostructures, and alloy nanocrystals, and this diversity potentiates the increase of the mass specific activity (MSA) of the nanoparticle while also minimizing the cost by using precious metals only in the surface of the particles. Thus, in order to attain a better control on shape, size and composition of the BM nanoparticles, it is critically important to understand the correlation between their structures and other properties [7–12]. Features expected for these BM nanostructures include the tuning of physical and chemical interactions among different atoms and phases that lead to novel functions, the changed miscibility and interactions unique to nanometer dimensions, and the morphological variations that are related to new particles [13].

Pt-based nanoparticles are frequently studied because of their high reactivity with organic molecules, which makes them capable of converting them to CO_2_ easily, and useful for electrocatalysis in fuel cells. There is an increasing interest in combining morphology engineering with the synergistic effect of adding a second metal to produce Pt-based particles with higher catalytic activities than pure Pt catalysts [14–17]. The stability at high temperatures and resistance against both physical impacts and chemical attacks make the Pt group metals quite distinguishable from other transition metals. Particularly, by combining Pt with secondary metals such as Ni, Co, Cu, Fe or Ti, it has been possible to produce particles with enhanced electrocatalytic performance towards the oxidation of CO [18–19], methanol oxidation reactions (MOR) [20–24], polymer electrolyte membrane fuel cells (PEMFCs) [15,25–28], hydrogen storage [29–30], and detecting hydrogen [31]. For instance, Wu et al. [32] studied a series of Pt-based bimetallic (Pt–Co, Pt–Fe, Pt–Ni, Pt–Pd) nanocrystals with octahedral and cubic shape and examined their facet-dependent catalytic performance for the oxygen reduction reaction (ORR). Guo and co-workers [33] synthesized FePt and CoPt nanowires by organic-phase decomposition and demonstrated that these systems are good catalysts for the ORR. Yun and co-workers [34] developed a unified embedded atom model to investigate the most energetically favorable atomic arrangements of Pd–Pt, Cu–Pt, Au–Pt and Ag–Pt nanoalloys using Monte Carlo simulations, obtaining intermetallic compounds for the Pd–Pt system, onion-like structures for the Cu–Pt system, and core–shell structures for Au–Pt and Ag–Pt. Yu et al. [35] investigated the formation of and dealloying of CuPt bimetallic nanoparticles in presence of hexadecylamine or PVP as capping agents, obtaining different morphologies of nanoparticles depending on their sizes. Recently, several groups have worked on the synthesis of CuPt core–shell and alloys nanoparticles, obtaining morphologies such as nanotubes, cubes, spheres, hollow structures and others [36–39]. These particles exhibit excellent catalytic activities for CO oxidation, methanol oxidation, formic acid electro-oxidation, and ORR, in comparison with other Pt-based nanoparticles [40–42].

In this paper, we describe the synthesis of monodispersed sub-3 nm Cu–Pt BM nanoclusters, and their characterization by spherical aberration (*C*_s_)-corrected scanning transmission electron microscopy (STEM) techniques. The use of high angle annular dark field (HAADF-STEM) images, intensity profiles, and energy dispersive X-ray spectroscopy (EDX) line scans, allowed us to study the atomic positions of Cu and Pt, and to compare the structure of the particles with the results of atomistic simulations. We applied a novel simulation method to study the growth mechanism of CuPt bimetallic nanoclusters; in particular, we explored the attaching of Pt atoms on Cu seeds by using grand-canonical Langevin dynamics (GCLD) simulations, which shows the formation of alloy structures in good agreement with empirical evidence.

## Experimental

### Chemicals and materials

Reagent-grade chemicals from Sigma-Aldrich such as chloroplatinic acid hydrate (H_2_PtCl_6_·*x*H_2_O, 99.9%), copper(I) acetate (CuCO_2_CH_3_, 97%), tetraoctylammonium bromide (TOAB, 99%), hexadecyltrimethylammonium bromide (CTAB), sodium borohydrate (NaBH_4_), 1-dodecanethiol, toluene and ethanol were used as received without further purification.

### Preparation of Cu–Pt bimetallic nanoclusters

In a first step, the H_2_PtCl_6_·*x*H_2_O metal ions were transferred into a toluene solution by a phase transformation process. An aqueous solution of 30 mM of 5 mL Pt precursor was mixed with a 60 mM of 10 mL solution of tetraoctylammonium bromide (TOAB, 99%) by vigorously stirring for 15 min. The organic phase was separated and formed 15 mM concentration stock solution.

In a second step, 0.03 g of copper(I) acetate and 0.240 g of hexadecyltrimethylammonium bromide (CTAB) were added into the 10 mL of toluene, and the mixture was heated at 120 °C under magnetic stirring to form a dark green solution. Afterwards, a freshly prepared sodium borohydrate (NaBH_4_) (72 μL, 4.6 M) was added under vigorously stirring. The dark green solution changed into dark brown within a minute indicating the formation of Cu nanoparticles. Then 36 μL of 1-dodecanethiol was added to stabilize the Cu nanoparticles. After preparing the Cu nanoparticles, 3 mL of the Pt precursor solution was quickly added into the colloidal solution and after 5 min of waiting NaBH_4_ (72 μL, 4.6 M) was added and the heating was stopped. The colloidal solution was left for cooling at room temperature, and the product was separated by centrifugation and washed with ethanol three times. The final product was redispersed in a toluene organic solution.

### Electron microscopy characterization

The morphology of the nanoparticles was characterized by transmission electron microscope (TEM) and high resolution transmission electron microscopy (HRTEM) by using a JEOL 2010F operated at 200 kV. The STEM images were recorded in a *C*_s_-corrected JEOL JEM-ARM 200F operated at 200 kV. HAADF STEM images were obtained with a convergence angle of 26 mrad and collection semi-angles from 50 to 180 mrad. The probe size used was about 0.09 nm with the probe current of 22 pA. In addition, bright field (BF) STEM images were recorded by using a collection semi-angle of 11 mrad. Energy dispersive X-ray spectra were obtained by using a probe size of 0.13 nm with a probe current of 86 pA.

### Models and simulation method

The simulation method employed is a grand-canonical Langevin dynamics (GCLD). Langevin dynamics is a method that extends molecular dynamics to represent the effect of perturbations caused by friction and eventual collisions occurring due to the presence of a solvent (the molecules in real systems are seldom under vacuum). For doing so, it makes use of stochastic differential equations, adding two terms to Newton’s second law to approximate the effects of neglected degrees of freedom. On the other hand, temperature can be controlled, approximating the canonical ensemble. Although it does not fully represent an implicit solvent (electrostatic screening and hydrophobicity), it mimics the viscosity of the medium. The original GCLD method was developed by M. M. Mariscal and co-workers [43–44] to study metallic deposition phenomena on crystalline planar surfaces. Now, the method has been extended to non-planar systems, like clusters and bimetallic nanoparticles (NPs). The simulation cell contains two distinct regions: the NPs region, and a much larger solution region. The solvent is not modeled explicitly, but it is provided as a stochastic bath. Thus, the solution part contains only metal particles that can either be of the same element as the metal atoms of the nanoparticle or different from them. All solution particles move according to Langevin's equation:


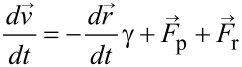


where γ is the friction constant, 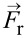 represents the random force acting on each particle and 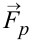 represents the force due to the potential interaction between the particles and the NP, as they do not interact with each other. The friction constant γ and the random force 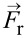 are related by the fluctuation–dissipation theorem. The Langevin dynamics was implemented by Ermak's algorithm [45]. Specifically, NP atoms interact with each other through potentials calculated from the embedded atom method [46]. To mimic the interaction between the NP atoms and the implicit solvent, they move according to Langevin's equation but with a friction coefficient that decreases as the bond order of the metal atoms increases, since atoms inside the NP (higher coordination number) are not expected to interact with the solvent as much as the atoms in the surface (lower coordination).

Following the experimental evidence, fcc structures were selected as Cu seeds for Pt growing. In particular we have employed the truncated octahedron (TO), the surface of which holds six square (100) faces and eight equilateral hexagonal (111) faces. TO structures of two sizes (*n* = 201 and *n* = 586) were used as seeds, which correspond to a diameter of 1.6 and 2.4 nm respectively. The simulations were carried out with our custom-developed code at 393 K (120 °C, as the experimental condition). 1 × 10^7^ LD steps were employed for each production run, giving a total simulation time of 20 ns.

## Results and Discussion

### Experimental results

**Figure 1:**
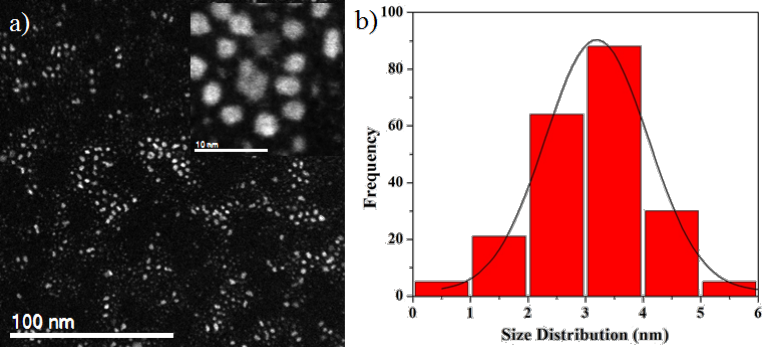
(a) STEM image of Cu–Pt bimetallic nanoparticles. The inset in (a) shows the HAADF-STEM image, and (b) Size distribution histogram, the average diameter is 3.0 ± 1.0 nm.

Figure 1a shows a representative STEM micrograph of as-synthesized Cu–Pt nanoclusters prepared by using the one-pot sequential reduction process described in a previous section. The inset shows a magnified HAADF-STEM image, illustrating the size and distribution of particles. The Cu–Pt nanoclusters were highly monodispersed and had an average size of 3.0 ± 1.0 nm. The size distribution histogram is presented in Figure 1b.

**Figure 2:**
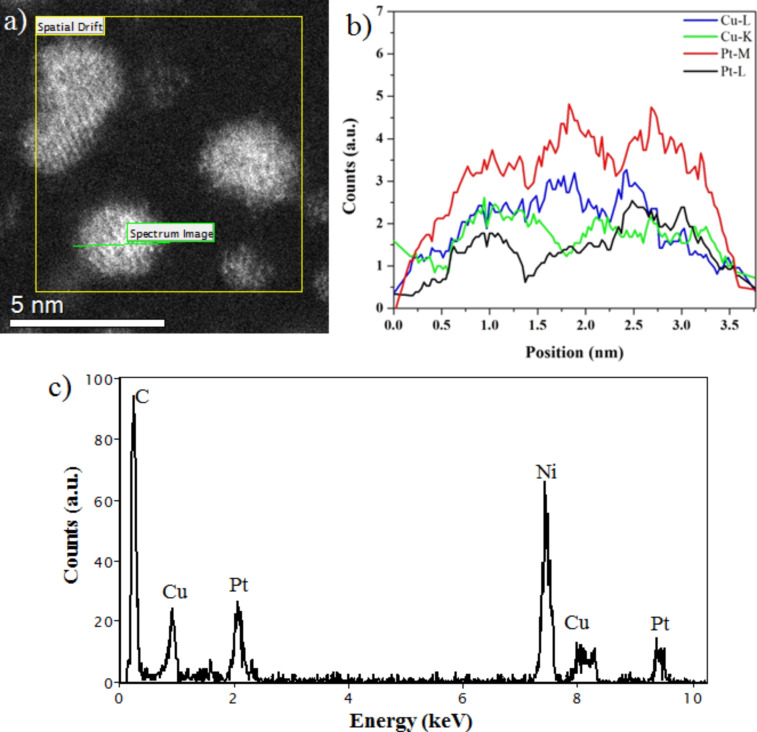
(a) HAADF-STEM image of Cu–Pt bimetallic nanoparticles, (b) Cu and Pt elemental line profiles along the green line across the nanostructure in (a), and (c) energy dispersive X-ray spectroscopy (EDX) spectrum of corresponding Cu–Pt bimetallic nanoparticles.

Figure 2a shows the HAADF-STEM image of Cu–Pt bimetallic nanostructures. The distributions of Cu and Pt in the nanoparticles were studied by EDX using the STEM mode. The EDX technique was applied to obtain 2D elemental mapping and cross-sectional compositional line profile of the nanostructures [47–48]. Here we observe the Cu–Pt bimetallic nanoalloy clusters and the compositional distribution of each element. Figure 2b (and Figure S2 in Supporting Information File 1) shows the EDX line profile of Cu and Pt, measured through the center of an individual nanoparticle (marked by a green line in Figure 2a). Both the Cu and the Pt signals were clearly traced across the entire particle (ca. 3 nm). Furthermore, the EDX spectrum also indicates that Pt atoms are present also in the surface region.

**Figure 3:**
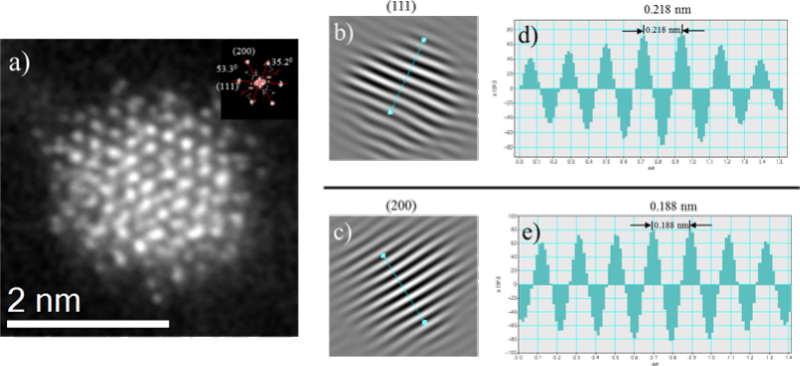
High resolution HAADF-STEM image of (a) Cu–Pt bimetallic nanoparticle; the inset represents the fast Fourier transform (FFT) of the image. The FFT was used to measure the interplanar distances corresponding to (111) (b, d) and (200) (c, e) planes.

Figure 3a shows a HAADF-STEM micrograph of Cu–Pt nanoparticles, in which we can clearly observe different atomic contrasts, related to the presence of Cu and Pt atoms. The inset of Figure 3a shows the corresponding fast Fourier transform (FFT). From the FFT it can be concluded that the crystal structure is fcc, and the zone axis in this case is [011]. Figure 3b was built considering only (111) reflections, so the fringe spacing corresponds to the interplanar distance for the (111) planes. Figure 3d shows the intensity profile corresponding to the line drawn perpendicular to the planes in Figure 3b. The measured interplanar distance is 0.218 nm, which compares well with the expected spacing of (111) planes of a CuPt alloy (0.219 nm) [35]. Figure 3c represents the filtered image considering only (200) reflections. Here the measured interplanar distance (shown in Figure 3e) was 0.188 nm.

**Figure 4:**
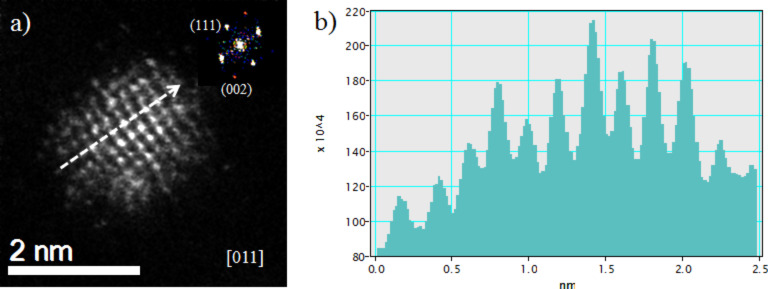
(a) *C*_s_-corrected STEM-HAADF image of a bimetallic Cu–Pt nanoparticle oriented along the [011] zone axis, and (b) intensity profile (in arbitrary units) measured over the white arrow marked in (a). For atomic columns of the same height, the differences in intensities are due to local changes in elemental composition.

HAADF-STEM imaging can be used to investigate the elemental distribution in bimetallic particles, under the assumption that the height of the atomic columns is fairly uniform, or that the differences in height are known, since the intensity signal depends strongly on the atomic number (*Z*). Figure 4a shows an atomic resolution STEM image of a Cu–Pt bimetallic nanoparticle oriented along the [011] zone axis, with crystal facets defined by the (111) and (002) planes. It can be easily noted that the intensity of two neighboring columns, likely to have roughly the same height, may have very different intensities. The intensity profile shown in Figure 4b, corresponding to the white line marked in Figure 4a, shows how different these intensities may be. Under the assumption of equal heights, these differences would be due to the local differences in the elemental composition of the atomic columns. As it will be shown in next section, our simulations predict similar intensity profiles.

### Simulation results

**Figure 5:**
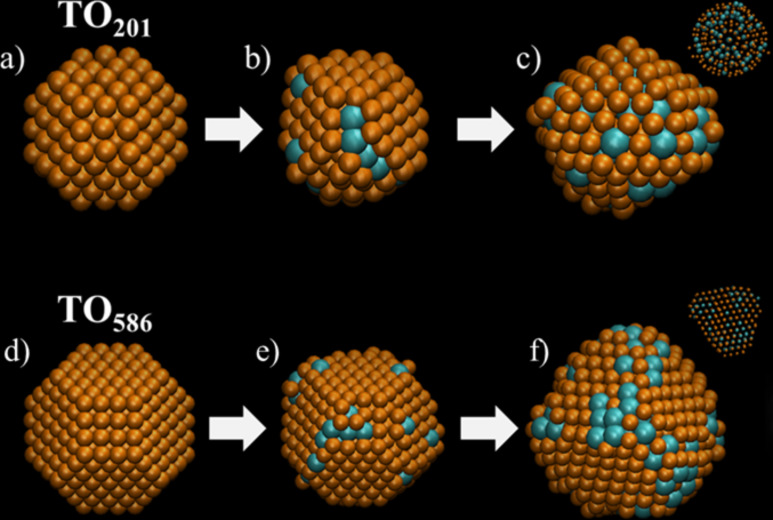
Snapshots taken during time evolution (20 ns) of Pt (cyan) deposition on the Cu NPs (orange) at *T* = 393 K: (upper panel) TO_201_ seed and (lower panel) TO_586_ seed. Atoms in solution are not shown for clarity.

Several grand-canonical Langevin dynamics (GCLD) simulations were performed in order to explore the growth mechanism of Pt atoms on Cu seeds. Figure 5 shows selected snapshots taken during time evolution of the deposition of Pt atoms on Cu nanoclusters with fcc TO morphology at different sizes. At first view, the simulation results predict structures very similar to those observed in the experiments. For the TO_201_ seed, the final diameter of the CuPt nanoalloys was ca. 1.8 nm, whereas for the TO_586_ seed it was ca. 2.6 nm. Even though this is slightly smaller than the diameters in the experiment (ca. 3 nm), it is within the expected value considering that if dynamics are allowed longer time the nanoparticle continues to grow. It is noteworthy that the TO_201_ Cu seed exhibits a structural transition from fcc to an icosahedron after 32–33 Pt atoms were deposited (Figure 5a–c). In the case of the TO_586_ Cu seed, the fcc structure is retained and in both cases CuPt alloys are evident, with an enrichment of Pt in the sub-surface layers. In all cases, the Pt deposition begins preferentially at the (100) faces due to the most favorable adsorption energy on open facets.

**Figure 6:**
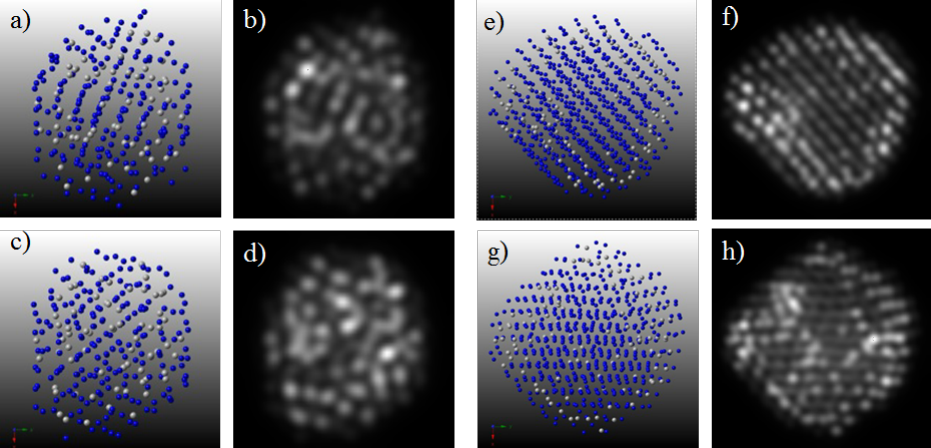
STEM simulated images of the final configurations shown in Figure 5. In (a–c) TO_201_ seed and (e–h) TO_586_ seed. The structures (c, d) and (g, h) were rotated by 30° around the y-axis. Note how the regions enriched in Pt appear brighter.

The final configurations of the GCLD simulations (Figure 5c,f), were used as input coordinates for the simulation of HAADF-STEM microscopy. For these simulations we used the multislice method as implemented in the xHREM package by Ishizuka, that uses an algorithm based on fast Fourier transforms. In Figure 6, the STEM simulation images corresponding to configurations Figure 5c and Figure 5f, respectively, are shown for two different orientations. It can be noted how the Pt-rich region of the nanoparticle brighter due to the *Z*-contrast feature of STEM. In addition, Figure S3 and Figure S4 in Supporting Information File 1 show the structures rotated by 30, 60 and 90° around the y- and x-axes, in which the Pt-rich regions appear brighter. These images are in very good agreement with the experimental images taken with the JEOL JEM-ARM 200F microscope.

In order to understand the novel structural transition observed for the Cu TO_201_ seed, the total energy of the nanoalloy was plotted against the number of GCLD steps (i.e., time) and compared with the number of Pt atoms added to the Cu seed (see Figure S5 in Supporting Information File 1). It is remarkable how the potential energy slightly increases just after adding 32–33 Pt atoms. Subsequently, while *N*_Pt_ = 33 constant, the potential energy decreases during the structural transition, a phenomenon itself which merits further research.

**Figure 7:**
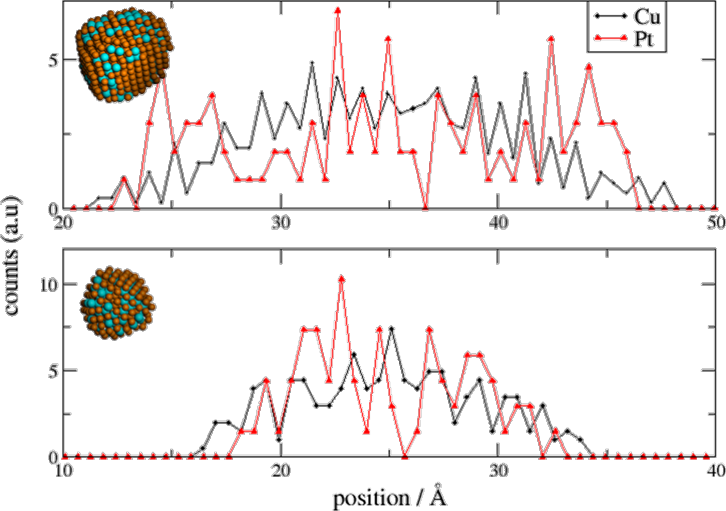
Cu and Pt elemental line profiles across the nanoalloy structure obtained after 20 ns of GCLD simulation, (upper panel) TO_586_ seed and (lower panel) TO_201_ seed.

To determine the mixing pattern of the obtained nanoalloys, we calculate the relative concentration of each atom type across the nanoalloys, a fingerprint that could be compared directly with the experimental results shown in Figure 2b. Based on the concentration profiles shown in Figure 7, it can be recognized that the resulting structures were alloyed NPs with an enrichment of Pt in the sub-surface layers.

To elucidate kinetic parameters, the mean square displacement (MSD) of the Pt atoms in the nanoalloy was calculated by taking previous configurations in the recorded trajectory as reference configuration. Moreover, the MSD reflects the relative change of diffusivity of the atoms at different temperatures and the activation energy (*E*_a_) for Pt diffusion in Cu NPs can be calculated, plotting the diffusion constant as a function of (*k*·*T*)^−1^ [49]. By means of a linear regression fit, an *E*_a_ of 0.009 eV/atom was found (Figure S6 in Supporting Information File 1). If we assume the transition state theory to be valid for the problem at hand, we can estimate the waiting time (*t*_w_) for a transition at 393 K to be equal to 1.30 ps. Evidently, this time is small and as a consequence we observe Pt diffusion inside the nanoalloy by means of atom dynamics simulations.

**Figure 8:**
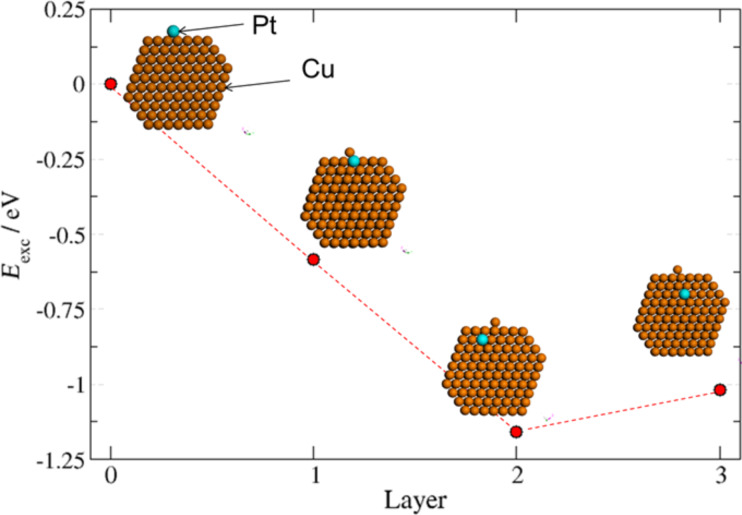
Calculation of exchange energy (E_exc_) for Pt atom diffusion in different sub-surface layers inside a Cu nanocluster with truncated octahedral geometry.

Figure 8 shows the change in the total energy (*E*_exc_) of the Cu nanocluster, when a Pt atom is exchanged with a Cu atom from the first, second and third sub-surface layers. The exchange energy was obtained through energy minimizations by using a conjugated gradient algorithm. *E*_exc_ is a thermodynamic parameter that indicates the stability of one structure with respect to another. Since *E*_exc_ < 0 for the inclusion of Pt atoms, the formation of alloy CuPt NPs is expected from energetic considerations.

## Conclusion

In summary, monodispersed bimetallic Cu–Pt alloy nanoparticles with highly uniform size and composition were synthesized by using a facile approach. Spherical aberration-corrected STEM, in combination with high resolution spectral and chemical analysis, has allowed us to study the atomic structure of the Cu–Pt bimetallic nanoclusters, and the chemical compositions of the particles were measured by STEM-EDX analysis. HAADF-STEM imaging allowed us to study the distribution of Cu and Pt, and to compare these results against atomistic simulations and simulated STEM images.

By using GCLD simulations, we have been able to study the formation mechanism of Cu–Pt bimetallic nanoclusters. In general terms alloyed nanoclusters were obtained. The enrichment of Pt layers obtained during the dynamic simulations could be easily explained by the activation energy for diffusion of Pt atoms on Cu. Both, thermodynamic and kinetic parameter (exchange energy and activation energy) confirms the existence of alloyed CuPt nanoparticles. The morphologies obtained with the simulations are in good agreement with the experimental findings.

## Supporting Information

File 1 Additional experimental data.
