# Supplementary material for: Synthesis, characterization, and growth simulations of Cu–Pt bimetallic nanoclusters
Source: Beilstein J Nanotechnol. 2014 Aug 27;5:1371–9. doi: 10.3762/bjnano.5.150 (PMC4168864; doi:10.3762/bjnano.5.150)
Supplement: File 1 — Additional experimental data. [file Beilstein_J_Nanotechnol-05-1371-s001.pdf]

Supporting Information

for

Synthesis, characterization, and growth simulations of Cu–Pt bimetallic nanoclusters

Subarna Khanal[1], Ana Spitale[2], Nabraj Bhattarai[1], Daniel Bahena[1], J. Jesus Velazquez-Salazar[1], Sergio Mejía-Rosales[3], Marcelo M. Mariscal*[2] and Miguel José-Yacaman*[1]

Address: [1]Department of Physics and Astronomy, University of Texas at San Antonio, One UTSA Circle, 78249, San Antonio, Texas, USA; [2]INFIQC, CONICET, Departamento de Matemática y Física, Facultad de Ciencias Químicas, Universidad Nacional de Córdoba, (XUA5000) Córdoba, Argentina and [3]Center for Innovation and Research in Engineering and Technology, and CICFIM-Facultad de Ciencias Físico-Matemáticas, Universidad Autónoma de Nuevo León, San Nicolás de los Garza, NL 66450, México.

Email: Miguel José-Yacaman* - miguel.yacaman@utsa.edu;

Marcelo M. Mariscal* - marcelo.mariscal@conicet.gov.ar

Additional experimental data

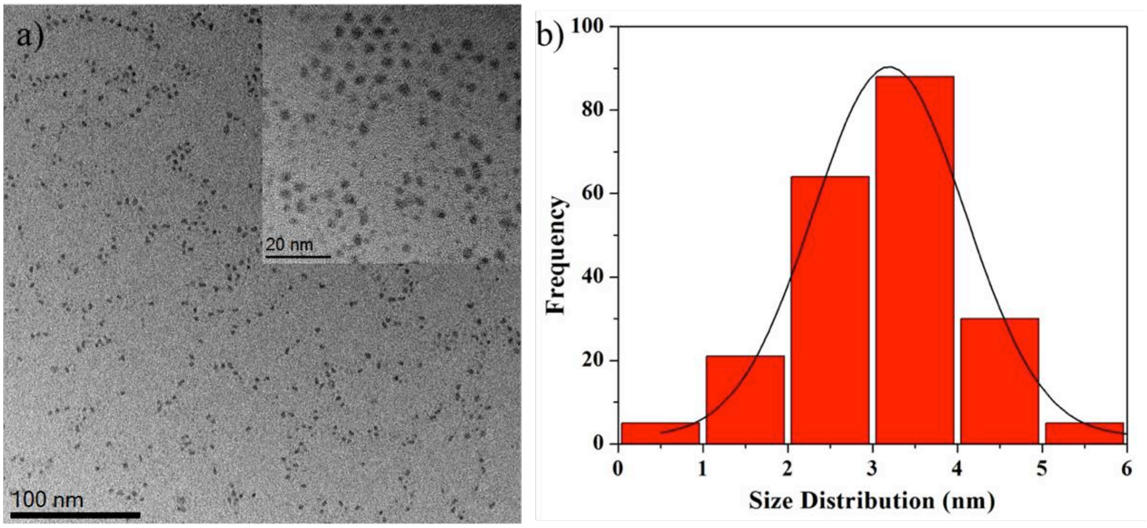

Figure S1: (a) Low magnification TEM image of Cu-Pt bimetallic nanoparticles. The inset in (a) shows the HTEM image, and (b) Size distribution histogram, the average diameter is 3 ± 0.5 nm.

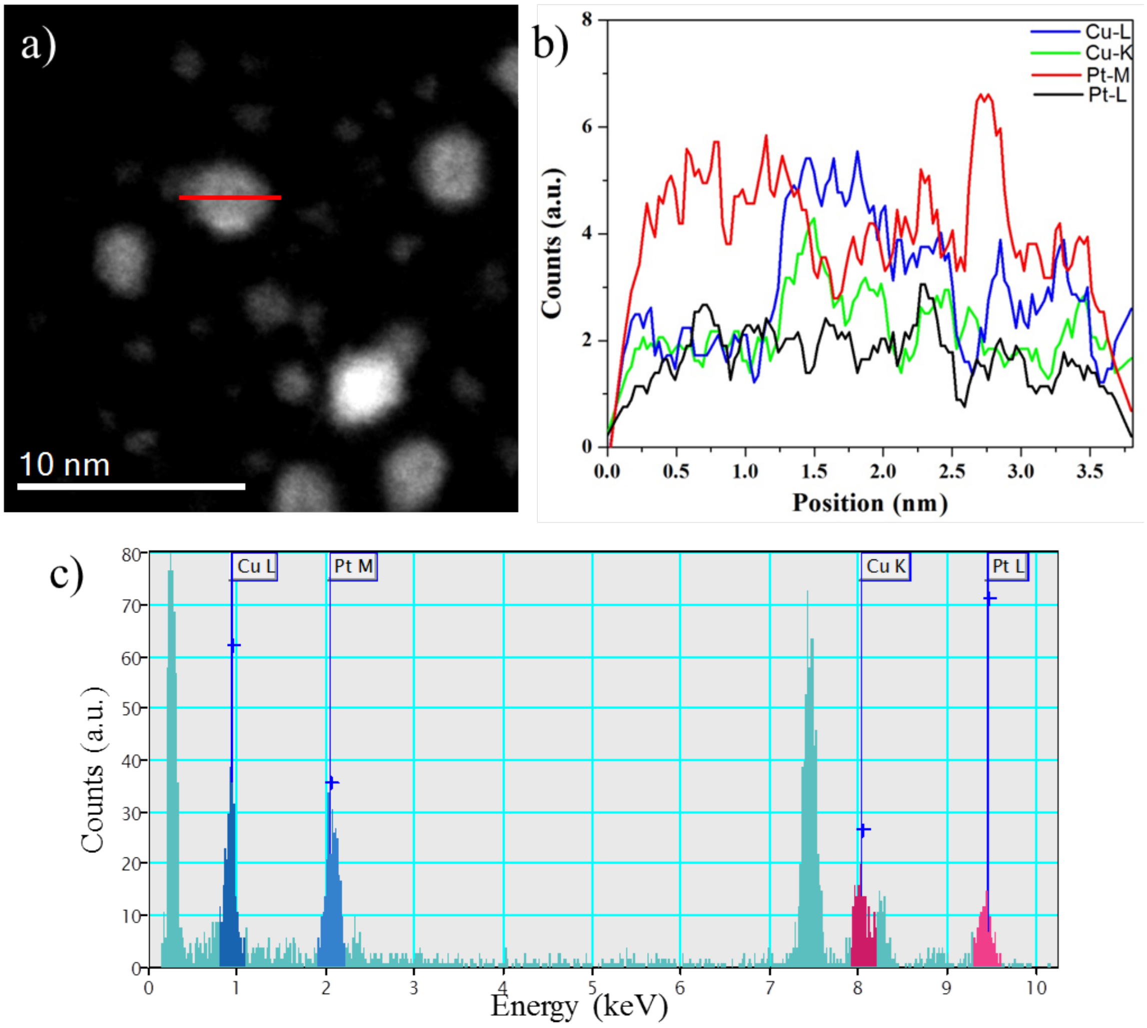

Figure S2: (a) HAADF-STEM image of Cu-Pt bimetallic nanoparticles, (b) Cu and Pt elemental line profiles along the red line across the nanostructure in (a), and (c) EDX spectrum of corresponding Cu-Pt bimetallic nanoparticles.

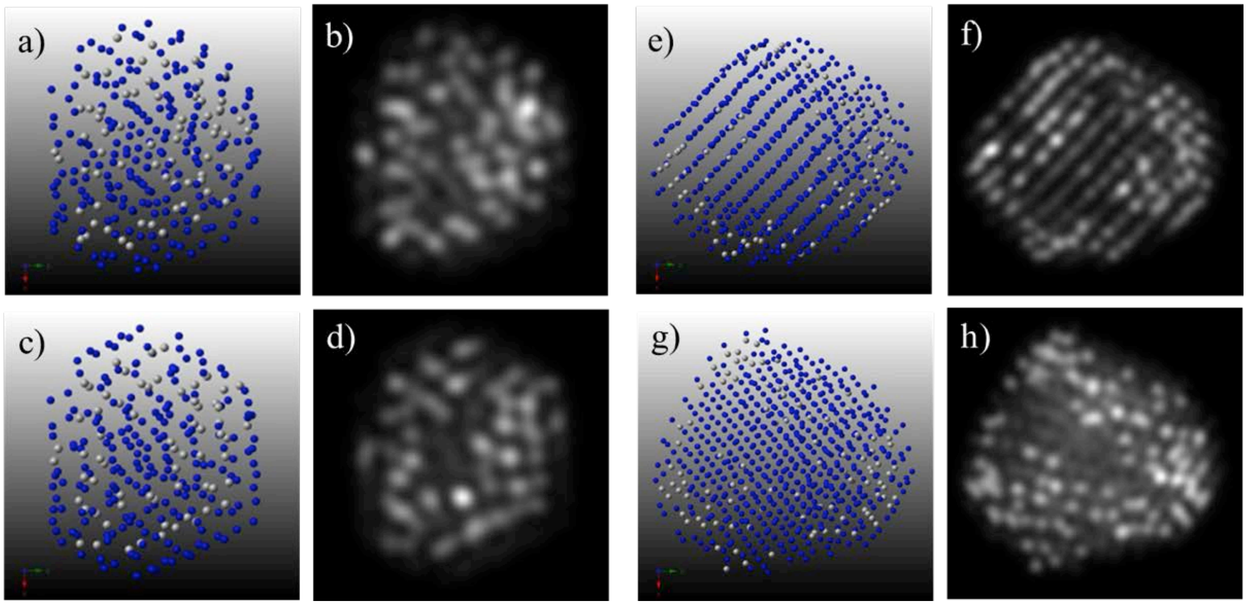

Figure S3: STEM simulated images of the final configurations shown in Figure 5. The structures (a, b) and (c, d) of TO_{201} particles were rotated 60° and 90° on y-axis. Similarly, the structures (e, f) and (g, h) of TO_{586} particles were rotated 60° and 90° on y-axis. Note how the regions enriched in Pt appears brighter.

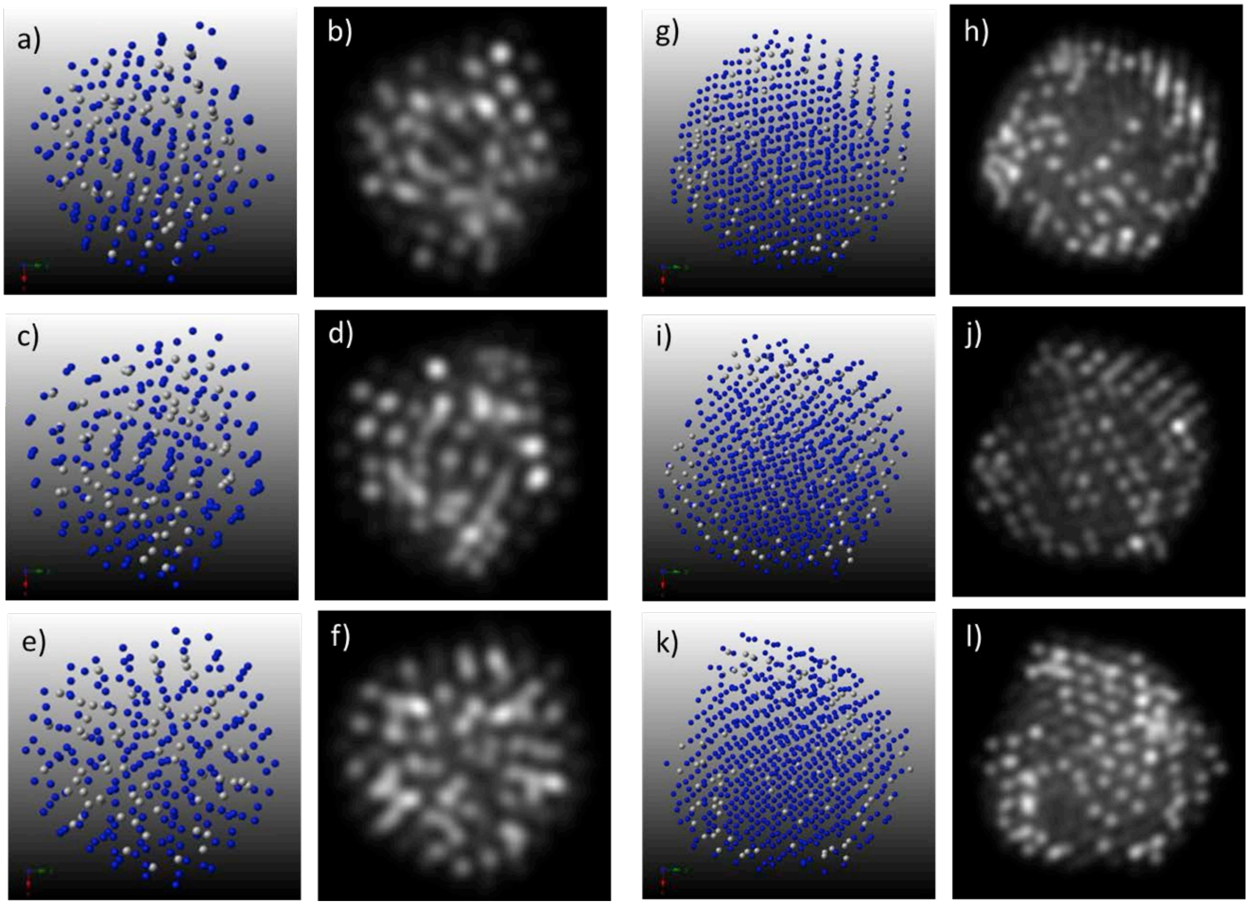

Figure S4: STEM simulated images of the final configurations shown in Figure 5. The structures (a, b), (c, d) and (e, f) of TO_{201} particles were rotated 30°, 60° and 90° on x-axis. Similarly, the structures (g, h), (i, j) and (k, l) of TO_{586} particles were rotated 30°, 60° and 90° on x-axis. Note how the regions enriched in Pt appears brighter.

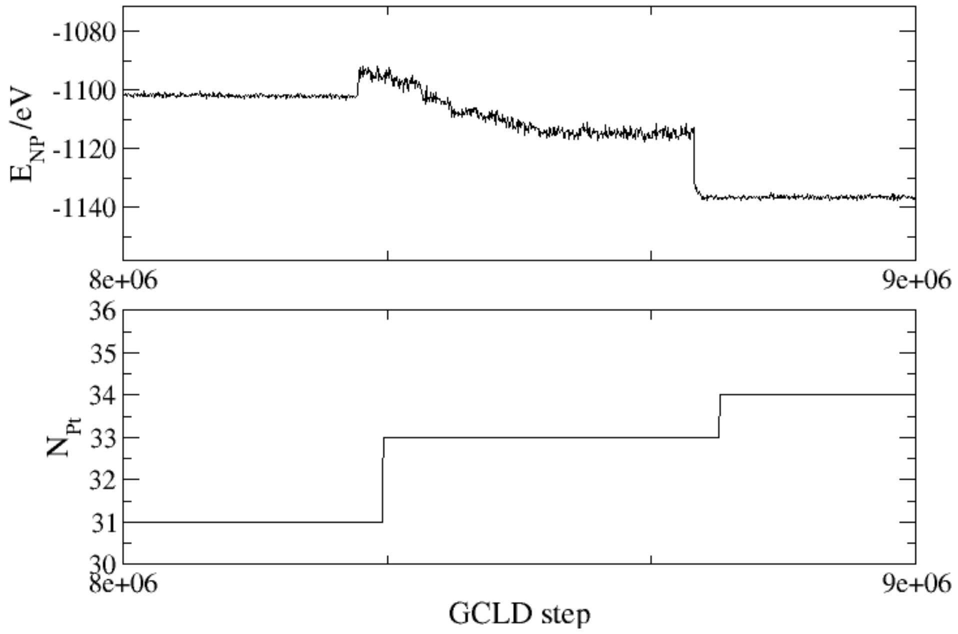

Figure S5: (top) Total energy of the nanoalloy as a function of GCLD steps, (lower) number of Pt atoms deposited on the Cu seed containing 201 atoms.

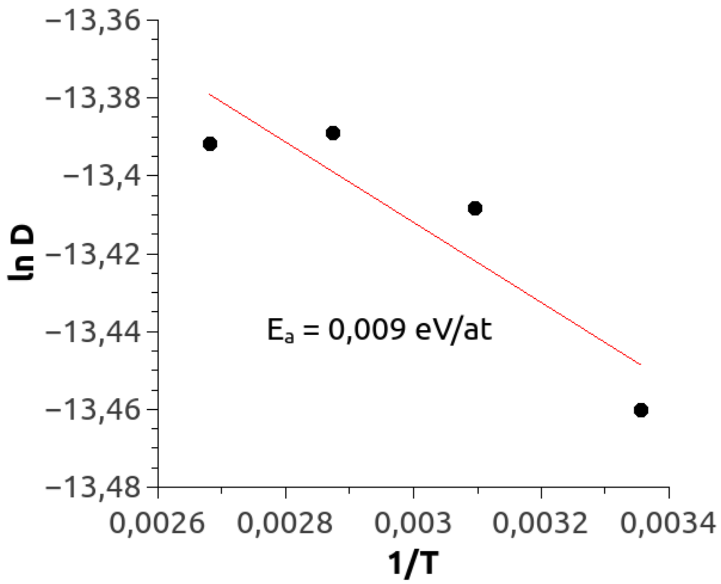

Figure S6: Diffusion coefficient calculated at different temperatures. From the linear regression fit, the activation energy for Pt diffusion in Cu is obtained.
